# Predictive Methodology for Quality Assessment in Injection Molding Comparing Linear Regression and Neural Networks

**DOI:** 10.3390/polym15193915

**Published:** 2023-09-28

**Authors:** Angel Fernández, Isabel Clavería, Carmelo Pina, Daniel Elduque

**Affiliations:** Department of Mechanical Engineering, University of Zaragoza EINA, María de Luna, 3, 50018 Zaragoza, Spain; iclaver@unizar.es (I.C.); carpina@unizar.es (C.P.); delduque@unizar.es (D.E.)

**Keywords:** injection, simulation, recycled, quality prediction, linear regression, neural network, polypropylene

## Abstract

The use of recycled polypropylene in industry to reduce environmental impact is increasing. Design for manufacturing and process simulation is a key stage in the development of plastic parts. Traditionally, a trial-and-error methodology is followed to eliminate uncertainties regarding geometry and process. A new proposal is presented, combining simulation with the design of experiments and creating prediction models for seven different process and part quality output features. These models are used to optimize the design without developing additional time-consuming simulations. The study aims to compare the precision and correlation of these models. The methods used are linear regression and artificial neural network (ANN) fitting. A wide range of eight injection parameters and geometry variations are used as inputs. The predictability of nonlinear behavior and compensatory effects due to the complex relationships between this wide set of parameter combinations is analyzed further in the state of the art. Results show that only Back Propagation Neural Networks (BPNN) are suitable for correlating all quality features in a single formula. The use of prediction models accelerates the optimization of part design, applying multiple criteria to support decision-making. The methodology is applied to the design of a plastic support for induction hobs. Furthermore, this methodology has demonstrated that a weight reduction of 27% is feasible. However, it is necessary to combine process parameters that differ from the standard ones with a non-uniform thickness distribution so that the remaining injection parameters, material properties, and dimensions fall within tolerances.

## 1. Introduction

Manufacturing of polymers using injection molding has been in use since the early 1950s. The benefits of this process are well known in terms of productivity and free shapes design, etc. [1]. Recent research has been focused on developing new technologies based on injection molding, such as gas-assisted injection molding [2], low-pressure technologies [3], and multi-material injection molding [4], among others, and other research has focused on the optimization of the quality of product and process [5]. Traditionally, quality features of injection molding technology can be roughly classified into three categories: (i) the dimensional and mechanical properties of the part, (ii) the adequacy of processing parameters to volumetric homogeneity of the parts, indicating how well the parts have been packed during injection molding, and how low warpage or thermally induced residual stresses are expected, and (iii) quality of the robustness of the process.

In order to achieve optimization purposes, from the point of view of quality, productivity, or mold design, simulation techniques have been used since the 1970s [6] apart from experimental trial-and-error methodology. Nowadays, the support of simulation techniques is essential in the design and development process of thermoplastic parts using injection molding carried out in four key stages of the design procedure. The first key stage is the feasibility analysis; the second one is when finishing the part design and mold pre-project, which is known as design for manufacturing. The third stage is during mold development, and finally, it arrives at the stage of continuous improvement during series production [7].

Despite the benefits of simulation, different reasons make results extrapolation difficult, among which the following reasons stand out: the complexity of thermal distribution and fluid flow [8], or the injection molding process and the nonlinearities affecting its physics [9,10], handling of incomplete data, and noise due to external uncertain factors. The interrelations between process parameters and design features, well known as design for manufacturing, forced the simulations of each geometry modification, becoming a highly engineering time-consuming task [11].

Therefore, the need for a multi-criteria design optimization approach to fit the quality requirements arises because any modification in process parameters, part thickness, or material properties will have consequences on process results, dimensions, weight, or part quality. The final solution should come from a combination or even superposition of the individual effects of the different parameters, making it almost impossible to predict the result if each simulation is not run step by step. This method implies a time-consuming engineering task requiring new procedures to achieve multi-criteria optimization, such as predictive models.

Injection Molding (IM) simulations are beneficial for plastic part designers because they predict the qualities and properties of components. They provide results about the process (temperatures, pressures), part homogeneity (volumetric shrinkage, residual stresses), and part quality (weight and control dimensions). Inputs are mainly part geometry and process parameters. They require both computational resources and expertise.

Machine Learning (ML) is a subset of artificial intelligence (AI) that focuses on developing algorithms and models that allow computers to learn and make predictions or decisions from data without being explicitly programmed. With Machine Learning (ML), it is possible to generate a predictive model of the results based on a limited number of Injection Molding (IM) simulations. The use of Machine Learning (ML) requires expertise but accelerates the process simulation, speeding up 1000 or more times the acquisition of desired results after prediction model generation. Back Propagation Neural Networks (BPNN) are Machine Learning processes. They are trainable with the results of a set of simulations. Then, they can predict these results using complex functions. Prediction models allow for the generation of newer simulation results without performing additional and complete simulations that take much time. BPNNs are used in relevant research, as shown below.

Artificial neural network (ANN) [12,13], genetic algorithm (GA) [14,15], regression methods [16,17], Taguchi experimental design method [18], and fuzzy [19] are the most preferred predictive and optimization methods found in the literature. ANN is an advantageous method for predicting linear and non-linear systems that has been widely used for modeling and prediction purposes in many fields [17]. The back propagation neural network is an algorithm that modifies the ANN so that numerical procedure is easier to evaluate and less computationally expensive, and it has the powerful ability of non-linear interpolation [13,20].

In the field of injection molding processes, most of the literature refers to prediction models where a single output variable is predicted. Many researchers have focused their efforts on predicting and controlling the behavior of injection machines [21,22] or nozzle positions [23] by managing the machine’s hydraulic system. The literature presents models to predict a dimension of the component based on general regression neural networks (GRNN) [24], ANN combined with GA [25], response surface methodology [26], or multiple linear regression [27,28]. Most of the studies in the literature focus on some parameters directly related to dimensions, such as shrinkage [12,18,29] and mainly warpage [30,31,32,33,34,35]. Weight is another indicator of part quality, and so some prediction models have been developed using genetic neural fuzzy [11], non-linear partial component regression [17], and, more recently, transfer learning procedures [36,37]. Some attempt has also been made to predict short shot [38] or weld lines [39,40], taking as input parameter gate location. Other research has been conducted to develop prediction models related to material parameters such as mechanical properties [41,42,43,44], fiber orientation [45], or even the selection of the thermoplastic material itself by transfer learning [46]. Regarding output related to process conditions, less research is found in the literature. Some authors have developed predictive models for barrel temperature [47,48] or cavity temperature during the cooling stage [49,50], but hardly any research is documented related to filling cavity pressure or melt front flow temperature, which are two of the most outstanding quality process control result, easily measurable and also related to some part defects.

As previously stated, injection molding is a very complex process in which different process parameters are interrelated and affected by important nonlinearities. That is why it is relevant to develop a predictive model considering multi-variables not only as inputs but also as predictable outputs. Despite this relevance, hardly any research is found considering multi-output predictable models. An approach is made in [51], where six different linear dimensions corresponding to a product design are considered as outputs. Similarly, other studies work with a set of dimensions as predicted values by means of a multilayer perceptron neural network [52] and a multi-output support vector regression [16]. These approaches all refer to dimension values as predictable results and do not include any other kind of result. Other research obtains results for multi-output parameters, but they implement different methods to convert the multi-objective optimization problem into a single objective evaluation problem through the entropy weight method [53] or analytic hierarchy process [54]. Although [55] works with three different outputs, this research focuses on the different Design of Experiments (DOE) strategies applied to obtain the training database rather than the outputs themselves. On the other hand, most of the results found in the literature come from an analysis that only considers process parameters such as temperatures, times, or packing pressure as inputs for the predictive models, but hardly any consider mold design features as input [29]. Despite the importance of thickness as the main geometric feature of an injected component, it is hardly considered in any reference as an input parameter.

The results could then be applied to the optimization of the design of a plastic support for induction hobs.

Therefore, a methodology for the simultaneous obtention of seven multi-output quality features related to material, process, and part quality from eight input variables, including process and mold design parameters, such as part thickness and flow leader thickness, is proposed based on the creation of prediction models using simulation results as training data. In order to find out what the most accurate mathematical model for prediction is, a comparison between multi-variable and multi-objective linear regression and back propagation neural network is shown. In the case of BPNN, different architectures are implemented, comparing their performance when predicting one or all outputs in a single architecture and comparing different numbers of neurons in the hidden layer.

## 2. Methodology

The primary aim of the new methodology is to enhance computational efficiency by simulating numerous cases, leading to the development of numerical prediction models for the results. During this phase, the developer’s involvement is minimal and mainly focuses on optimizing the outcomes based on these predictions. The simulation of the cases predicted as optimal is deferred until the final stage.

**Figure 1:**
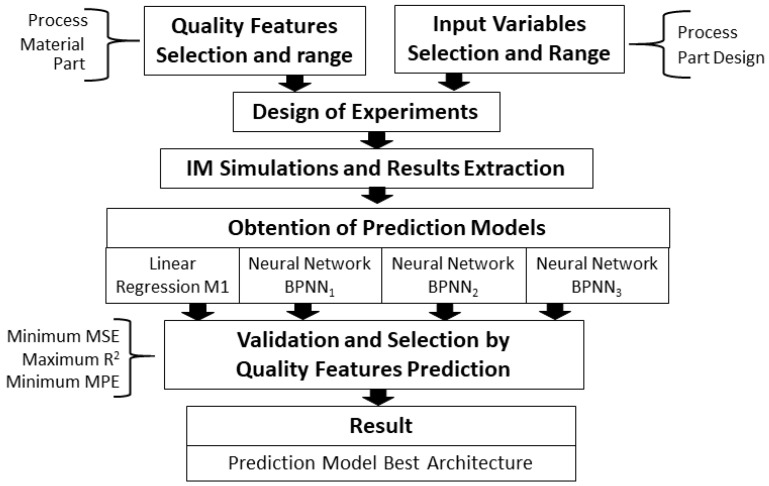
Flow chart of the methodology.

The methodology includes the following steps (Figure 1):

Next, a comprehensive explanation will be given of how to apply this new methodology to improve a recycled polypropylene part manufactured through injection molding.

For a better understanding of this methodology, it will be applied to a specific case, which involves the design of a plastic support—a component with which the authors already have experience. Previous research [56,57,58,59] has demonstrated the feasibility of replacing fiber-reinforced polyamide with talc-filled polypropylene and subsequently with recycled polypropylene. The current part thickness is 1.8 mm. A feasibility study focused on weight reduction by thinning the part. However, reducing its main thickness below 1.5 mm has proved unachievable. Therefore, the new methodology aims to minimize the weight even further. To achieve this objective, an experimental study will be conducted to reduce the thickness of the plastic support to 1.0 mm. This will be accomplished by employing thicker flow leaders to ensure proper filling of the piece while ensuring optimal quality for all other manufacturing parameters.

### 2.1. Quality Features and Input Parameters Selection: Design of Experiments

**Figure 2:**
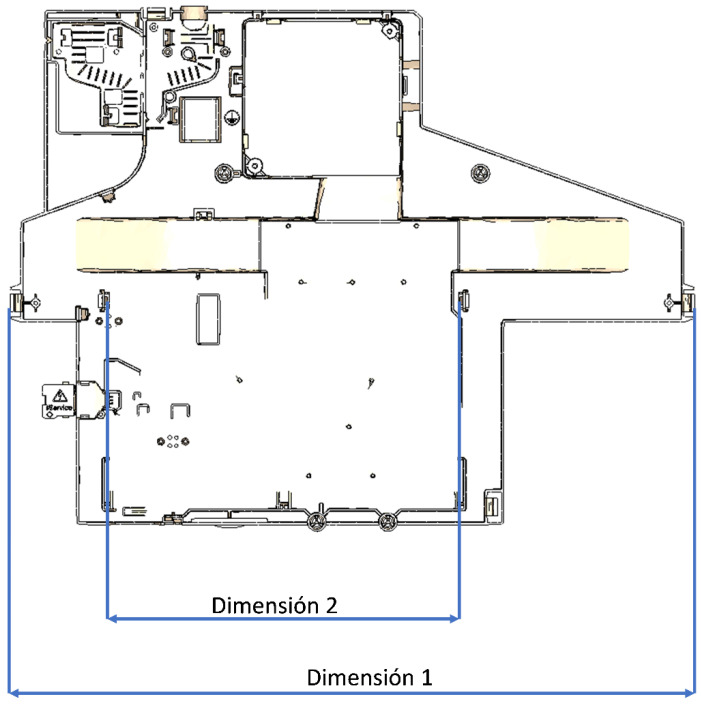
Control dimensions.

A selection of seven parameters has been applied to quantify the quality of the part design. These dependent variables are named quality features, as they are the results whose outcome should ideally remain within an admissible range. The simultaneous in-depth analysis of them is a holistic approach to understanding the total quality as the process encompasses process, material, and detailed design. Two features are related to the quality of the process: Minimum flow front temperature indicates the uniformity of melt flow front speed and surface quality, while maximum injection pressure indicates the robustness of the manufacturing process and short shot prevention. The next two are related to the adequacy of processing parameters to volumetric homogeneity of part: maximum volumetric shrinkage is an indicator of homogeneity of plastic packing along the cavity, while average distortion of warpage presence is due to residual stresses. All these features are related to a recycled homopolymer polypropylene filled with 40% talc. The last three are direct characteristics of the final part, which must fit the tolerances requirement: Total part weight is an indicator of plastic savings and potential environmental impact reduction. Dimensions 1 and 2 are shown in Figure 2. Dimension 2 corresponds to the distance between the two snap fits. Dimension 1 corresponds to the part width.

The admissibility range of each feature used during the optimization process is detailed in Table 1. The range of admissibility for process results is based on the researcher’s previous experience. Researchers have collaborated broadly with the injection molding industry, and ranges regarding material and process are normal in the manufacturing industry. Other ranges, such as weight and dimensions, are requirements of the Original Equipment Manufacturer (OEM). The minimum flow temperature should not be below the no-flow temperature of the polymer, and the maximum injection pressure should not exceed the actual processing one. Volumetric shrinkage and average linear distortion should fit a suitable value according to the plastic grade properties. Variations below ±8% are imposed. Finally, small variations in weight (2%) and dimensions (<0.1%) are within product tolerances.

The objective of this research is to obtain the predictive model that best fits the outcome of quality features.

A set of eight dependent variables (from A to G) is defined. They have been carefully chosen from among all the potential influential variables that can be controlled during the part design phase. These include six processing parameters and two geometry features. The most relevant process parameters influencing the quality results of the injected part are filling time, melt and mold temperature, post-pressure time and pressure, and cooling time. The main geometry features are the nominal thickness of the part, which determines the final part weight, and the flow-leaders thickness, which improves the filling flow pattern and filling of the cavity. The range of variation selected for the input variables is wide enough to simulate the main defects on the part, as will be described in Section 3.1 Simulation results. A wide range of variations has also been defined to ensure that there will be outcomes within and outside the range defined in Table 1. All parameters shown in Table 2 are feasible and fit with the recommended values for the plastic material.

Injection molding simulations are time-consuming. A design of experiments is needed to minimize the total simulation runs. Using a fractional factorial design [60] or a Plackett–Burman design [21] increases the number of runs by a power of two or four, respectively, so we should need 128 runs for eight parameters. A 2^k−p^ = 2^8−3^ = 32 runs fractional factorial design could be performed according to [60], but not all effects would be considered. If we want to estimate all the main effects and two-way interactions and afford eight parameters for errors, 38 runs would be needed following the D-optimal criterion [61,62]. The D-optimal design of experiments is shown in Table 3.

### 2.2. Injection Molding Simulation and Results Extraction (Cadmould Software)

Cadmould v15.0 (2022), an injection molding simulation tool, has been coupled with the D-optimal screening design to enhance the simulation process outcomes and quality feature extraction.

**Figure 3:**
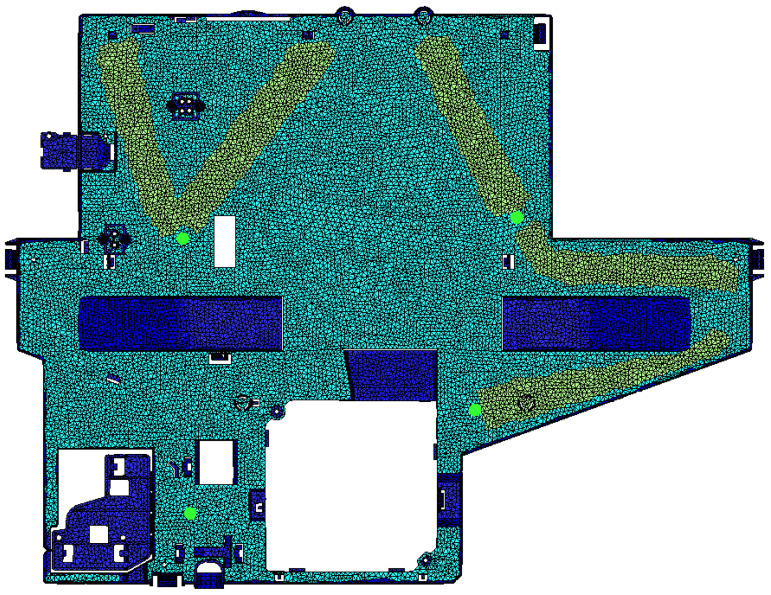
Part Model mesh, injection points (green circles), and Thickness groups for geometry variations design. Group 1 (dark blue) area of invariable thickness. Group 2 (light blue) is the largest area with nominal thickness. Group 3 (yellow) flow leaders are thicker than Group 2. Group 4 (red) has small and thin hinges.

The 3D part model is shown in Figure 3. Four different element groups have been defined to vary their thickness independently, as shown in Figure 3. Group 1 (dark blue) is an area of invariable thickness because it affects the thermal behavior of the electronic components assembled in the part. Group 2 (light blue) is the largest area of the part and includes the nominal thickness. Any variation in it implies nearly a proportional variation in total part weight, so it is one independent variable. Group 3 (yellow) is a set of flow leaders whose thickness is thicker than the nominal value of the part. Additionally, it is one of the variable inputs because it improves the advance of melt through. Group 4 (red) corresponds to small and thin hinges with specific functions that cannot be modified. Additionally, four injection points (green circles) have been selected.

A mesh is needed for Finite Element Method calculations. Cadmould uses a surface model with triangles along the inner and outer surfaces of the part, joined by 1D vectors. These vectors point from the center of gravity of triangles to the opposite surface and are normal to them. The thickness of the part is the length of these vectors. Mesh details are described in Table 4. Local parallel plate flow is simulated using the generalized Hele–Shaw-Approximation [63]. A study of mesh independence has been developed, decreasing the mesh size until the distortion remains nearly constant. As a result, a mesh size with a side length of the elements below 3.5 mm (2.957 mm average) has been selected. This means the element size is below 0.519% of part dimensions (0.248% average), and a total number of 1,557,575 calculation points are created.

The material selected for the simulation is a recycled homopolymer polypropylene filled with 40% talc. The grade is E-RIALFILL H 07 40 T, and the supplier is Rialti S.P.A. The main properties are extracted from the Camould database.

Carreau–WLF model is used to determine the influence of temperature and shear rate on melt viscosity (Equation (1)) [64,65,66].

(1) $$\eta =\frac{a_{T}\times P_{1}}{{(1+a_{T}\times P_{2}\times \dot{\gamma )}}^{P_{3}}}\;\;\;\;where\;\log\left(a_{T}\right)=\frac{8.86\times \left(T_{0}-T_{2}\right)}{101.6\times \left(T_{0}-T_{2}\right)}-\frac{8.86\times \left(T-T_{2}\right)}{101.6\times \left(T-T_{2}\right)}$$

IKV–Schmidt model is used to determine the influence of temperature and pressure on specific volume (Equations (2)–(4)) [67].

(2) $$v=\frac{PS_{1}}{PS_{4}+p}\;+\;\left(\frac{PS_{2}}{PS_{3}+p}\right)\times T\;\;\;\;\;\;\;\;\;\;\;\;\;\;\;\;\;\;\;\;\;\;melt\;range$$

(3) $$v=\frac{PF_{1}}{PF_{4}+p}+\left(\frac{PF_{2}}{PF_{3}+p}\right)\times T+PF_{5}\times e^{\left(PF_{6}\times T-PF_{7}\times p\right)}\;\;\;\;solid\;range\;\;\;$$

(4) $$v=PK_{1}+PK_{2}\times p\;\;\;\;\;\;\;\;\;\;\;\;\;\;\;\;\;\;\;\;\;\;\;\;\;\;transition\;range$$

### 2.3. Obtention of Multivariate Linear Regression Prediction Model

The inputs and outputs of the injection molding simulation previously performed following the D-optimal screening design are used to create regression models. These models have been created using R, version 4.0.5 (31 March 2021). The interface used is R-commander 2.7.2. [68,69].

The regression model follows this formula (Equation (5)) [70]:

OUTPUT_i_ = a_i0_ + a_i1_.A + a_i2_.A^2^ + a_i2_.B + a_i3_.B^2^ + a_i4_.C + a_i5_.C^2^ + a_i6_.D + a_i7_.D^2^ + a_i8_.E + a_i9_.E^2^ + a_i10_.F + a_i11_.F^2^ + a_i12_.G + a_i13_.G^2^ + a_i14_.H + a_15_.H^2^ + a_i16_.A.B + a_i7_.A.C + a_i18_.A.D + a_i19_.A.E + a_i20_.A F + a_i21_.A.G + a_i22_.A H + a_i23_.B.C + a_i25_.B.D + a_i6_.B E + a_i27_.B F + a_i28_.B G + a_i29_.B.H + a_i30_.C.D + a_i31_.C.E + a_i32_.C.F + a_33_.C.G + a_i34_.C.H + a_i35_.D.E + a_i36_.D.F + a_i37_.D.G + a_i38_.D.H + a_i39_.E.F + a_i40_.E.G + a_i41_.E.H + a_i42_.F.G + a_i43_.F.H + a_i44_.G.H (5)

where A to H are the input variables (see Table S1) and a_ij_ are the coefficients for each output and term. Coefficients a_i0_ denote the intercept terms. OUTPUT_i_ refers generically to output variables (see Table 1). In this research, the subscript “i” will take values from 1 to 7. OUTPUT_1_ represents the value prediction that the multivariable linear regression model obtains for the flow front temperature, OUTPUT_2_ for the maximum injection pressure, and so on up to OUTPUT_7_.

### 2.4. Obtention of Artificial Neural Network Prediction Model

The inputs and outputs of the injection molding simulation are used to train and test different back propagation neural networks (BPNN). These fitting models have been created using the Neural Net Fitting app embedded in MATLAB R2022a for academic use (9.9.0.1467703) [71].

BPNN has been chosen because they are very precise to fit nonlinear problems. These artificial neural networks’ architecture consists of:

- Input, a vector with the independent variables.
- Input layer (I_i_), where the components of the inputs vector are normalized between [−1,1];
- Hidden layer, where weights and bias are applied to inputs and sigmoid neurons (LW_i_) create newer inputs for the next layer;
- Output layer, where weights and bias are applied to hidden layer outputs and linear neurons (OW_i_) generate the outputs between [−1,1];
- Output, where a prediction vector is obtained after denormalizing the output layer;

Three BPNN architectures have been selected from among different options. BPNN_1_ is an 8 × 7 net with 8 sigmoid neurons connected to all inputs and bias in the hidden layer and 7 linear neurons connected to all hidden neurons and bias in the output layer. BPNN_1_ is the most straightforward design to predict 7 outputs from 8 independent inputs, considering certain similarities in the nonlinear behavior of the predictions. The main advantage of BNN_1_ design is the prediction of all outputs with just one complex function. BPNN_2_ is an 8 × 1 net with 8 sigmoid neurons connected to all inputs and bias in the hidden layer and just one linear neuron connected to all hidden neurons and bias in the output layer. BPNN_2_ is an accurate design to predict one output from 8 independent inputs considering the nonlinear behavior of this relationship. Different BPNN_2_ weights matrices and bias vectors have been developed for each output, similarly to the case of linear regression. The advantage of BPNN_2_ is the prediction of outputs independently of each other with an increased precision even above regression. BPNN_3_ is a 24 × 7 net with 24 sigmoid neurons connected to all inputs and bias in the hidden layer and 7 linear neurons connected to all hidden neurons and bias in the output layer. BPNN_3_ is more complex than others. It is possible with it to predict 7 outputs from 8 independent inputs with just one function, decreasing the cross-influence between outputs and considering nonlinear behavior with more complex functions. The main advantage of BPNN_3_ is the increased correlation between all inputs and outputs simultaneously. Other architectures, such as fewer neurons in the hidden layer, have been discarded because of the lack of benefits in the results. Additionally, the increase of up to 2 hidden layers has been discarded because the complexity of the fitted model lays further the focus of this research as it increases the computational and results post-processing effort. Any increment in the number of neurons in the hidden layer improves the results of the cases described in this section.

All BPNNs have been trained with the data of 38 injection molding simulations and tested afterward with the rest described in Section 3.2. The algorithm used is Bayesian Regularization. It offers better results than Levenberg–Marquardt or Scaled Conjugate Gradient with a small input data set. Although the calculation time is greater, the small number of experiments makes it negligible. All BPNN have been retrained until the Mean Squared Error (MSE) has been minimized and R-Squared (R^2^) maximized simultaneously for training and testing experiments. Epochs, the number of iterations, is limited to 1.000, and these finish when the adaptive weight is minimized. Expected gradients are close to 1.00 × 10^−7^ in the best cases (see Tables S2–S7 for all BPNN_i_ bias and weight values).

### 2.5. Validation: Prediction Model Selection by Quality Features Prediction

Once predictive models are obtained, they are used to test the effects of new values for the inputs on the quality features. This research conducts numerous additional predictive cases by using the four models. Additionally, subsequent Injection Molding (IM) simulations are developed to compare their results with the predictions.

These additional IM simulations are helpful in determining which prediction model is superior. The results of each quality feature of each new case are compared, and the squared error and percentage error are calculated. For each quality feature, MSE, MPE, and R2 are calculated. MSE and R^2^ for each quality feature are the metrics to compare prediction models. Minimum MSE and maximum R^2^ are used to decide which prediction model is the best.

One field of application is the development of a design for a manufacturing project. In this case, additional results for any combination of values of input parameters can be obtained with the selected prediction models without additional injection molding simulations. This is a time-saving process if the prediction model is good enough. The results of using the predictive model will allow for informed decision-making regarding improvements in design and manufacturing conditions.

## 3. Results and Discussion

### 3.1. Injection Molding Simulation Results

In the design of thermoplastic injected components, it is customary to opt for a uniform thickness guided by a conservative criterion. The approach to minimizing the component’s weight involves a combination of thin thicknesses across the majority of the piece, combined with small, thicker areas that facilitate proper filling. Simulation of the injection process highlights that this decision carries an inescapable risk. The occurrence of incomplete filling or warpage, alongside other defects, becomes more likely. Consequently, a more comprehensive investigation into these defects’ underlying causes becomes imperative to predict and prevent their appearance.

**Figure 4:**
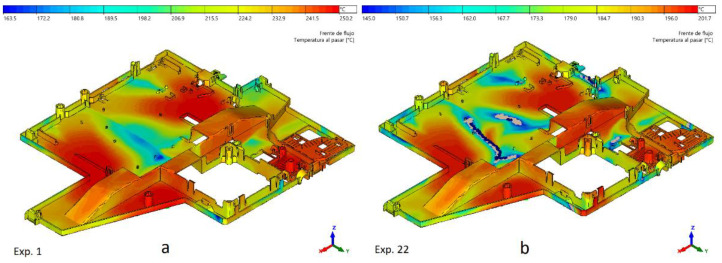
Flow front temperature for experiments 1 (**a**) and 22 (**b**) and short shot appearance.

Short shots can arise from various causes. One of them involves the incorrect selection of process parameters. A minimum flow front temperature (Tmin) below 135 °C could be dangerous to complete the cavity filling. Additionally, maximum pressure (Pmax) should not overcome the upper limit. Figure 4 shows the flow front temperature under normal conditions in Experiment 1 (exp. 1) and the lack of plastic in critical areas (gray holes) under bad process conditions (exp. 22).

**Figure 5:**
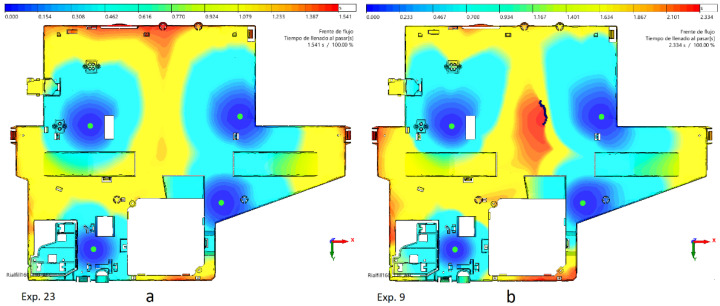
Filling pattern of experiments 23 (**a**) and 9 (**b**).

Short shots can also appear as the result of poor part design. The difference in thickness between the thinnest piece and the thickest flow leaders can cause a lack of filling due to the hesitation effect. Figure 5 shows the difference in filling patterns between cases 23 and 9 due to the hesitation effect. Identical processing temperatures and speed are applied, but the appearance of air traps produces unfilled areas. Short shots can occur even with high injection and mold temperatures if the injection speed is not as fast as in case 9. Blue represents the first areas being filled, and red represents the last ones. Filling of the cavity in exp. 23 is completed, as the thickness difference is only 0.3 mm. However, in exp. 23, it grows up to 0.7 mm. The flow advances slower in the center of the part, developing a large red area where the border drawn by black spots reveals air traps and unfilled areas.

Maximum volumetric shrinkage (Vshrk) and average linear distortion (Distor) are results mostly influenced by injection molding temperature and post-filling pressure. The analysis of the results (Table 5) reveals that extreme conditions such as high temperature or low pressure increase both values and opposite input parameters decrease them. The greater the volumetric shrinkage or average distortion, the greater the possibility of sink marks or warpage appearing. A lower value of both would be associated with a highly residual stressed part. Properly combining process parameters will lead to the desired intermediate outcomes for these variables. However, this combination does not precisely align with the one that prevents defects such as short shots, thus necessitating subsequent optimization. For example, in case 22, outcomes have admissible values of 12.5% volumetric shrinkage and 0.876% average distortion but show a short shot. The same happens for cases 15 and 17.

Although the results described so far serve to control a quality process, the objective with the weight of the part is its minimization. Obviously, the minimum weight values are reached with the thinnest piece and leaders. The representative thickness of the component is provided in the flat area and is originally 1.8 mm. The expected weight is approximately 432 ± 1 g. Achieving a uniform reduction in this thickness allows for the design of a feasible component at 1.5 mm, resulting in a target weight of 372 ± 1 g. This research aims to ascertain the feasibility of combining overall thicknesses between 1.0 and 1.2 mm with flow leaders ranging from 1.5 to 1.7 mm to achieve a minimum weight. Outcomes go from 313 g of case 31 to 365.4 g of case 6. However, in many cases, they are associated with poor results from the rest of the variables that basically would imply physical restrictions that would prevent the feasibility of the part. Part weight is also slightly influenced by melt temperature and post-injection pressure. Variations of about ±2 g depending on process conditions are simulated, so posterior optimization work is needed.

Finally, Dimensions 1 (Dim1) and 2 (Dim2) are key factors for the dimensional quality of the part and correct assembly in the mechanical system. Both dimensions should vary in the same direction with changes in the injection parameters; however, this is not the case. The value of Dimension 2 varies mainly with thermo-volumetric changes, so any decrease in temperature or increase in pressure will increase its value. In the second, distortion has more influence. Distortion is inherent to the injection molding process and is increased by residual stresses, which also increase with decreasing temperature or increasing pressure. As the increase in distortion implies a shortening of Dimension 1, the opposite effect happens with respect to Dimension 2.

**Figure 6:**
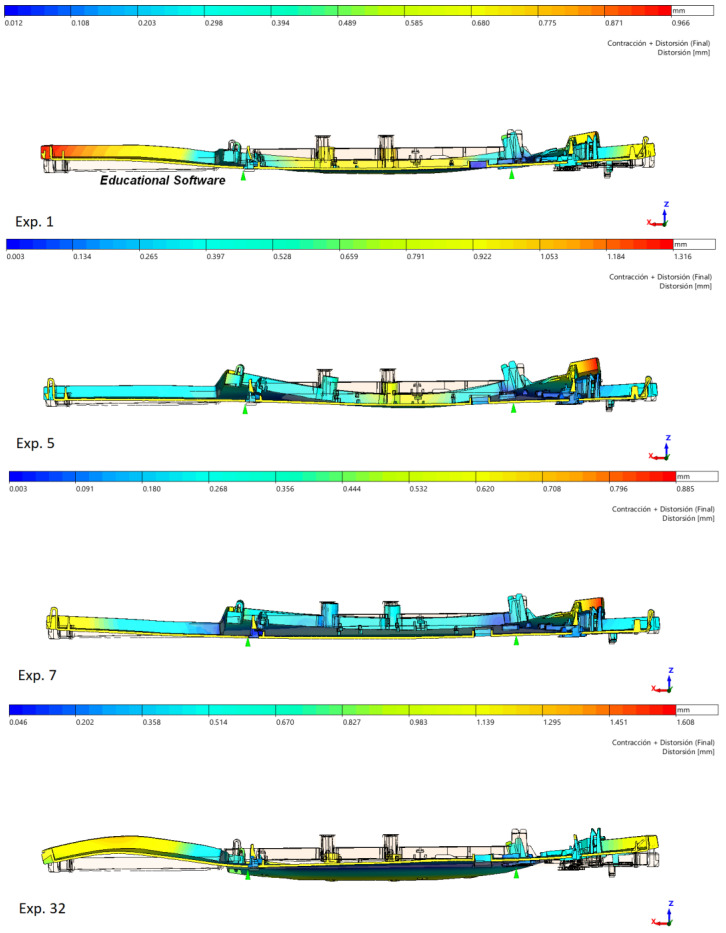
Distortion and deformed shape at Dimensions 1 and 2 for experiments 1, 5, 7, and 32.

The result of these opposing effects can be seen in Figure 6. A cross-section of the part distortion through Dimensions 1 and 2 is represented for Experiments 1, 5, 7, and 32. The rest of the experiments follow one of these shapes, more or less. Experiment 1 (top) simulates the recommended process parameters and should fit the dimensional tolerances. The deformed part exhibits different curvatures along the section, positive in the center (Dim.1) and negative in the extremes (Dim.2). Warpage of the part causes shortening of the final dimensional values compared with expected values. Experiment 32 (down) parameters are the highest mold temperature and post-injection pressure and should compensate for Exp.1 defects. Nevertheless, the part is designed with minimum thickness and thickest flow leaders, increasing the warpage and reducing Dimension 1 to 1.09 mm and Dimension 2 to 0.3 mm. Exp. 5 (middle top) simulates maximum melt and mold temperatures and should be smaller than Exp.1 or Exp.32. Part thickness is 1.2 mm, and flow leaders are 1.5 mm. More uniform thicknesses and higher process temperatures reduce the warpage significantly, and the part shows a straight cross-section with the longest Dimension 1, rather than expected, but some warpage reducing Dimension 2. Exp. 7 (middle down) exhibits a smooth continuous curvature with adequate dimensions compared to colder or less pressurized parts. This geometry is designed with the smallest thickness for both part and flow leaders. Minimum distortion allows Dimensions 1 and 2 to fit tolerances, and cavity volume is the minimum. However, process parameters differ from the recommended ones, so further optimization is needed again.

The quality features extracted from IM simulations run from the D-optimal design of experiments are shown in Table 5. These results and all the additional simulation results for testing and validation will be named targets for further analysis.

### 3.2. Prediction Models Comparison and Discussion

A linear regression model with both quadratic and mixed terms has been fitted for each output. Due to the screening design of experiments, all effects are included in the model so that all 38 residuals are 0, and there are no residual degrees of freedom. The multiple squared error, R^2^, is 1. Table S1 in Supplementary Data shows the predictive model’s coefficients a_ij_ for process parameters, part quality, weight, and dimensional results.

The predicted values for all 38 simulations are exactly the same as the simulation outcomes.

Three different architectures of back propagation neural networks have been fitted for the results. The results obtained for BPNN_1_ are shown in Tables S2 and S3 in the Supplementary Data. They show weight and bias terms between the input and hidden layers (Table S2) and weight and bias terms between the hidden and output layer (Table S3). Tables S4 and S5 show resultant terms for BPNN_2_, and Tables S6 and S7 for BPNN_3_.

A set of 114 additional simulations has been created to test the models, comparing their targets with the prediction outputs. The input parameters for these new simulations are within the range of the initial ones (Table 2), approximately dividing it by eight, as shown in Table 6. Only thickness variations remain the same.

R-squared measures the level of correlation for each output variable, while Mean Squared Error and Mean Percentage Error (MPE) are effective indicators of the precision. The comparison of the correlation (R^2^) and precision (MSE and MPE) of the predictions obtained with the different models is shown in Table 7, Table 8, Table 9, Table 10, Table 11, Table 12 and Table 13.

Linear regression shows a good correlation for all results (R^2^ > 0.899) except the maximum for volumetric shrinkage. BPNN_1_ shows a poor correlation for maximum volumetric shrinkage and average linear distortion and none for Dimensions 1 and 2. This lack of correlation is due to the fact that the prediction of the dimensions is more complex than that of the average shrinkage. Warping appearance is a consequence of the residual stresses, as explained in Section 2, and modifies the predictions of the dimensions from what is expected. Additionally, BPNN_1_ simultaneously combines the same results of hidden layer neurons in all output layer neurons, while linear regression estimates each output independently. The combination of these two factors demonstrates the need for a more complex neural network design to improve the results. Special consideration should be given to the prediction of the flow parameters (flow front temperature and max pressure), which is better with BPNN_1_. The reason is that they depend only on the mold-filling parameters and are quite independent of the rest of the inputs and outputs. The good correlation obtained for the prediction of the final weight of the piece is remarkable. The explanation is that the thickness variation is substantially more influential than the rest of the inputs. The analysis of the weights of BPNN_1_ ratifies this argument. Flow parameters and part weight predictions are mainly weighted by neurons LW07, LW04, and LW01, respectively. In turn, these are weighted mainly by the part thickness. The predominance of one variable over the others increases the correlation between inputs and output. The case of Dimensions 1 and 2 is very different. For them, the weighting is similar in all neurons, and no input predominates in its prediction model. This is an additional source for the lack of correlation.

Linear regression shows good precision for most of the results with high correlation. In the case of minimum flow front temperature and maximum pressure, their MPE is 2.65% and 5.55%, respectively. It seems not to be a high-accuracy prediction, but it could be enough for an early design stage during the part development process. For Dimensions 1 and 2, MPE seems to be better (0.032% and 0.017%), but it corresponds to a deviation around 0.170 mm and 0.047 mm, respectively, which is too high for tolerance analysis. In the case of BPNN_1_, only flow properties or part weight predictions show an acceptable precision.

BPNN_2_ significantly improves correlation and precision in all outputs. The reason is that each neural network is fitted to predict one output only, respectively. So, none of the other outputs influence the predictions. R^2^ goes from 0.99 for Dimension 1 up to 0.99999 for part weight. Analyzing in detail the output neurons, a similar weight from hidden layer neurons is seen within the range of 0.497–0.998. Significantly, the contribution of any input is not negligible in the prediction of Dimension 1. The precision is now more than acceptable for Dimensions 1 and 2. The results for MPE (0.0096% and 0.0036%) correspond to a deviation of around 0.0567 mm and 0.0099 mm, respectively, which is suitable for tolerance analysis.

Finally, BPNN_3_ corrects the insufficiencies observed in BPNN_1_ in terms of correlation and accuracy. It also improves those of linear regression. Correlation is achieved for all outputs where BPNN_1_ and regression could not. This is because it uses a greater number of sigmoidal neurons in the hidden layer, substantially reducing the interdependence on the output predictions. In contrast, the higher quality of the BPNN_3_ is achieved at the cost of a greater use of computational resources. R^2^ goes from 0.93946 for Dimension 1 to 0.99998 for total part weight. The precision is now slightly lower for Dimensions 1 and 2. The results for MPE (0.0241% and 0.0118%) correspond to a deviation of around 0.121 mm and 0.027 mm, respectively, which are accurate enough for tolerance analysis.

## 4. Conclusions

- A new methodology has been developed and tested for the early design of complex plastic parts, making it possible to predict features of the final part by combining the optimal design of experiments, process simulation, regression, neural network fitting, and prediction model generation and application.
- Results present different prediction models for seven required output features simultaneously related to material, part, and process quality. These models predict newer results in real time, varying eight input molding and part thickness design parameters without launching additional time-consuming simulations.
- Simulation has shown the near unpredictable part dimensions due to the combination of thermo-volumetric and residual stress effects. The results of these could be additive or compensatory and then modify the dimensions of the part. A trial-and-error simulation process based on rules of thumb cannot overcome this situation. For this reason, the creation and application of a predictive model accounting for these effects is the fastest way to achieve an optimal solution.
- A multivariable and multicriteria prediction model based on BPNN is recommended for the prediction model. The prediction model obtained from a BPNN with 24 sigmoidal neurons in the hidden layer (BPNN3) has shown the best precision and highest correlation when predicting all 7 quality features with just one function. This number of neurons is a multiple of 8 because the 8 inputs are the independent variables. It is also a multiple of 3 because even though there are 7 output features, they have been selected from three distinct fields: process, material, and part quality. The product of these two figures explains the sufficient number of neurons. Results obtained from this model allow better design options complying with the restrictions defined for the outputs. For a future research study, the authors propose developing an optimization algorithm based on the selected predictive model. Additionally, deep research is needed to include new criteria in this methodology, taking into account inputs from active agents in the decision-making process: designers, mold makers, and converters. Moreover, research has to focus on including high-level variables to make this knowledge and application transferable.
- The main novelty of the paper lies in the fact that prediction models can define seven different outputs simultaneously from eight different input parameters. Input parameters are related not only to process conditions but also to design features such as part thickness and flow leader thickness. Outputs are related to process, material, and part quality, such as cavity temperature and pressure during injection, volumetric shrinkage, distortion after ambient conditions are reached, or weight and critical dimensions of the part.

## Figures and Tables

**Table 1:** Quality features, range of admissibility for the optimization process.

Parameters	Objective	Minimum	Maximum
Min. Flow Front Temperature (°C)	160	−15	40
Max. Molding Pressure (Bar)	700	500	725
Max. Volumetric Shrinkage (%)	12.5	11.5	13.5
Average Linear Distortion (%)	0.9	0.83	0.97
Total Part Weight (g)	320	313	327
Dimension 1 (mm)	529.0	528.6	529.4
Dimension 2 (mm)	272.5	272.2	272.8

**Table 2:** Process parameter and geometry variations for factorial analysis.

Parameters	Lower	Center	Upper
A, Filling Time (s)	1.4	1.75	2.1
B, Melt Temperature (°C)	200	250	300
C, Cooling Time (s)	28	35	42
D, Mold Temperature (°C)	40	50	60
E, Post-pressure Time (s)	9.6	12	14.4
F, Post pressure (Bar)	320	400	480
G, Part Thickness (mm)	1.0	1.1	1.2
H, Flow Leaders Thickness (mm)	1.5	1.6	1.7

**Table 3:** D-optimal design of experiments for 8 parameters.

Experiment	A	B	C	D	E	F	G	H
1	1.75	250	50	12	400	35	1.1	1.6
2	2.1	300	60	14.4	480	28	1.0	1.7
3	2.1	300	60	14.4	320	42	1.0	1.7
4	2.1	300	60	14.4	320	28	1.2	1.7
5	2.1	300	60	9.6	480	42	1.2	1.5
6	2.1	300	40	14.4	480	42	1.2	1.7
7	2.1	300	40	14.4	480	42	1.0	1.5
8	2.1	300	40	14.4	320	28	1.0	1.7
9	2.1	300	40	9.6	480	42	1.0	1.7
10	2.1	300	40	9.6	480	28	1.2	1.5
11	2.1	300	40	9.6	320	42	1.2	1.5
12	2.1	200	60	14.4	480	28	1.2	1.7
13	2.1	200	60	14.4	320	42	1.2	1.7
14	2.1	200	60	14.4	320	28	1.0	1.5
15	2.1	200	60	9.6	480	28	1.0	1.5
16	2.1	200	60	9.6	320	42	1.0	1.5
17	2.1	200	60	9.6	320	28	1.0	1.7
18	2.1	200	40	14.4	480	42	1.2	1.5
19	2.1	200	40	14.4	480	42	1.0	1.7
20	2.1	200	40	14.4	320	28	1.2	1.7
21	2.1	200	40	9.6	480	42	1.2	1.7
22	2.1	200	40	9.6	320	28	1.0	1.5
23	1.4	300	60	14.4	320	42	1.2	1.5
24	1.4	300	60	9.6	480	28	1.2	1.7
25	1.4	300	60	9.6	320	42	1.2	1.7
26	1.4	300	60	9.6	320	28	1.0	1.5
27	1.4	300	40	14.4	480	42	1.0	1.7
28	1.4	300	40	14.4	320	28	1.2	1.5
29	1.4	300	40	9.6	480	42	1.2	1.5
30	1.4	300	40	9.6	320	28	1.2	1.7
31	1.4	200	60	14.4	480	28	1.0	1.5
32	1.4	200	60	14.4	320	28	1.0	1.7
33	1.4	200	60	9.6	480	42	1.0	1.7
34	1.4	200	60	9.6	320	28	1.2	1.5
35	1.4	200	40	14.4	480	42	1.0	1.7
36	1.4	200	40	14.4	320	42	1.0	1.5
37	1.4	200	40	9.6	480	28	1.0	1.7
38	1.4	200	40	9.6	320	42	1.0	1.7

**Table 4:** Mesh details.

Type of Element	Number of Elements	Number of Nodes	Averg. Element Area (mm^2^)	Averg. Side Length (mm)	Averg. Element Thickness (mm)
10-N tetrahedra	162,860	62,303	3.529	2.957	1.498

**Table 5:** Selected simulation results.

Experiment Number	Tmin (°C)	Pmax (Bar)	VShrk (%)	Distor (%)	Weight (g)	Dim1 (mm)	Dim2 (mm)
1	163.5	696.4	11.8	0.914	339.5	528.65	272.58
2	163.0	584.9	14.3	0.984	319.2	528.00	272.36
3	163.0	584.9	14.2	0.833	320.6	528.99	272.71
4	187.1	431.2	16.3	0.977	363.3	528.13	272.26
5	181.5	552.9	12.4	0.772	360.3	529.37	272.99
6	187.1	431.2	13.2	0.802	365.4	529.45	272.86
7	164.6	563.8	12.0	0.832	315.4	529.25	272.79
8	163.0	584.9	15.7	0.990	319.0	527.90	272.27
9	132.6	619.3	13.5	0.786	320.9	529.07	272.90
10	181.5	552.9	15.1	0.926	358.6	528.20	272.56
11	181.5	552.9	14.7	0.776	360.3	529.33	272.95
12	143.2	818.8	12.7	1.001	363.3	528.47	272.36
13	143.2	818.8	11.5	0.843	365.1	529.55	272.78
14	127.7	1081.8	12.7	0.986	313.8	528.21	272.36
15	127.0	1407.8	12.5	0.877	314.1	528.19	272.36
16	127.0	1407.8	11.6	0.760	315.6	529.09	272.71
17	127.9	1382.7	12.5	0.879	319.4	528.06	272.37
18	142.8	955.4	11.6	0.848	359.6	529.51	272.80
19	126.8	1019.8	11.5	0.838	320.5	529.33	272.73
20	143.2	818.8	12.5	0.993	363.3	528.50	272.38
21	130.0	908.7	11.5	0.805	365.3	529.51	272.91
22	127.0	1407.8	12.5	0.876	314.1	528.19	272.36
23	224.8	530.1	15.6	0.831	359.7	529.14	272.73
24	218.6	481.0	14.5	0.922	363.9	528.14	272.55
25	218.6	481.0	14.8	0.774	365.5	529.16	272.89
26	191.5	630.3	15.4	0.961	313.9	527.69	272.44
27	202.9	594.5	12.5	0.832	320.6	528.92	272.78
28	224.8	530.1	16.5	0.992	357.9	527.96	272.28
29	219.8	571.6	12.8	0.781	360.2	529.17	272.97
30	218.6	481.0	16.1	0.929	363.7	528.04	272.46
31	146.8	1030.4	12.7	1.054	313.2	527.18	272.24
32	148.7	1053.5	12.8	1.034	318.6	527.56	272.25
33	129.4	1121.8	11.7	0.831	320.4	528.65	272.78
34	156.6	1072.6	12.7	1.008	357.2	526.85	272.37
35	159.6	828.9	11.6	0.852	364.9	529.44	272.74
36	146.8	1030.4	11.5	0.886	314.8	529.24	272.69
37	129.4	1121.8	12.5	0.978	318.5	527.49	272.39
38	163.5	696.4	11.8	0.914	339.5	528.65	272.58

**Table 6:** Process parameters variations for testing simulations, Octiles (Oct).

Parameters	Oct.1	Oct.2	Oct.3	Oct.5	Oct.6	Oct. 7
A, Filling Time (s)	1.5	1.575	1.65	1.85	1.925	2
B, Melt Temperature (°C)	215	225	240	260	275	285
C, Cooling Time (s)	29.5	31.5	33	37	38.5	40.5
D, Mold Temperature (°C)	42	45	48	52	55	58
E, Post-pressure Time (s)	10.2	10.8	11.4	12.6	13.2	13.8
F, Post pressure (Bar)	340	360	380	420	440	460

**Table 7:** Correlation and errors for minimum flow front temperature.

Term	Regression	BPNN_1_	BPNN_2_	BPNN_3_
Mean Squared Error (MSE)	13.5456	7.74970	1.50553	0.03878
Mean Percentage Error (MPE)	2.64801	1.90270	0.80820	0.12848
R-Squared (R^2^)	0.982626	0.98730	0.99767	0.99993

**Table 8:** Correlation and errors for maximum molding pressure.

Term	Regression	BPNN_1_	BPNN_2_	BPNN_3_
Mean Squared Error (MSE)	1720.65	5.55311	2.11534	1.78237
Mean Percentage Error (MPE)	5.55165	0.29905	0.17997	0.19770
R-Squared (R^2^)	0.971839	0.99986	0.99994	0.99995

**Table 9:** Correlation and errors for maximum volumetric shrinkage.

Term	Regression	BPNN_1_	BPNN_2_	BPNN_3_
Mean Squared Error (MSE)	1.95318	0.98291	0.02160	0.05527
Mean Percentage Error (MPE)	10.1168	7.79060	1.12097	1.80582
R-Squared (R^2^)	0.296026	0.29579	0.98470	0.96033

**Table 10:** Correlation and errors for average linear distortion.

Term	Regression	BPNN_1_	BPNN_2_	BPNN_3_
Mean Squared Error (MSE)	0.00011	0.00517	1.3461 × 10^−5^	0.00022
Mean Percentage Error (MPE)	1.15390	8.12836	0.40936	1.72502
R-Squared (R^2^)	0.968651	0.060989	0.996164	0.940400

**Table 11:** Correlation and errors for total part weight.

Term	Regression	BPNN_1_	BPNN_2_	BPNN_3_
Mean Squared Error (MSE)	0.01159	0.27001	0.00471	0.00535
Mean Percentage Error (MPE)	0.03224	0.15546	0.02055	0.02160
R-Squared (R^2^)	0.999982	0.999438	0.999990	0.999989

**Table 12:** Correlation and errors for Dimension 1 (longer).

Term	Regression	BPNN_1_	BPNN_2_	BPNN_3_
Mean Squared Error (MSE)	0.02873	0.20477	0.00257	0.01626
Mean Percentage Error (MPE)	0.03210	0.08565	0.00960	0.02416
R-Squared (R^2^)	0.899864	0.240797	0.990435	0.93946

**Table 13:** Correlation and errors for Dimension 2 (shorter).

Term	Regression	BPNN_1_	BPNN_2_	BPNN_3_
Mean Squared Error (MSE)	0.00215	0.03412	9.8153 × 10^−5^	0.00105
Mean Percentage Error (MPE)	0.01700	0.06777	0.00363	0.01189
R-Squared (R^2^)	0.929677	0.019345	0.996713	0.964266

## Data Availability

Not applicable.

## Supplementary Materials

The following are available online at https://www.mdpi.com/article/10.3390/polym15193915/s1, Table S1: Predictive model coefficients for Min. Flow Front Temperature, Max. Molding Pressure, Max. Volumetric Shrinkage and Average Linear Distortion, part weight dimension 1 and 2., Table S2: Weight and bias between input layer and hidden layer (BPNN1)., Table S3: Weight and bias between hidden layer and output layer (BPNN1)., Table S4: Weight and bias between input layer and hidden layer (BPNN2) for Dim1 prediction., Table S5: Weight and bias between hidden layer and output layer (BPNN2) for Dim1 prediction., Table S6: Weight and bias between input layer and hidden layer (BPNN3)., Table S7: Weight and bias between hidden layer and output layer (BPNN3).
