# Supplementary material for: Predictive Methodology for Quality Assessment in Injection Molding Comparing Linear Regression and Neural Networks
Source: Polymers (Basel). 2023 Sep 28;15(19):3915. doi: 10.3390/polym15193915 (PMC10575229; doi:10.3390/polym15193915)
Supplement: Supplementary file 1 [file polymers-15-03915-s001.zip › polymers-2611307-supplementary.pdf]

polymers

MDPI

Supplementary Materials

Predictive Methodology for Quality Assessment in Injection Molding Comparing Linear Regression and Neural Networks

Angel Fernández *, Isabel Clavería, Carmelo Pina and Daniel Elduque

Department of Mechanical Engineering, University of Zaragoza EINA, María de Luna, 3 50018 Zaragoza, Spain; iclaver@unizar.es (I.C.); carpina@unizar.es (C.P.); delduque@unizar.es (D.E.)
* Correspondence: afernan@unizar.es; Tel.: +34-629786393

Table S1. Predictive model coefficients for Min. Flow Front Temperature, Max. Molding Pressure, Max. Volumetric Shrinkage and Average Linear Distortion, part weight dimension 1 and 2.

Term	Tmin	Pmax	VShrk	Distor	Weight	Dim1	Dim2
intercept	26.316827	4854.9032	23.645124	0.909752	327.1799	516.8223	271.96927
A	67.981888	691.2438	2.566796	−0.311249	3.2150	2.13932	0.00379
A^2	0	0	0	0	0	0	0
B	0.626692	−28.3975	−0.202042	0.003325	0.0346	0.04116	−0.00035
B^2	0.000617	0.0436	0.000578	−0.000008	0	0	0
C	−0.647687	−13.9774	0.050656	0.003574	0.0191	0.07299	-0.00370
C^2	0	0	0	0	0	0	0
D	−2.433563	−21.8872	0.907790	0.010438	−0.1431	−0.02904	−0.03198
D^2	0	0	0	0	0	0	0
E	−0.075673	−0.6696	0.012736	−0.000573	−0.0138	0.009653	0.00229
E^2	0	0	0	0	0	0	0
F	0.540182	8.6209	0.091325	0.001133	−0.0116	−0.05974	−0.00358
F^2	0	0	0	0	0	0	0
G	22.973892	−2202.8648	−4.546871	0.509376	−216.5400	−2.02030	0.48474
G^2	0	0	0	0	0	0	0
H	−31.182648	−1541.4846	−4.595055	−0.000874	30.3008	3.30289	0.79147
H^2	0	0	0	0	0	0	0
A.B	−0.364967	−1.1637	0.001567	0.000585	−0.0059	−0.00533	−0.00044
A.C	−0.219922	−6.9725	−0.022066	0.001610	−0.0119	−0.00729	0.00427
A.D	−0.471409	−1.5643	0.076590	0.001222	−0.0102	0.03840	0.00060
A.E	−0.006525	−0.0099	−0.002824	0.000104	−0.0010	−0.00203	−0.00015
A.F	0.074574	0.1136	−0.048057	−0.000572	−0.0038	0.01516	0.00034
A.G	−0.908029	−574.2546	−1.791098	0.196788	−0.8630	−0.54199	0.32004
A.H	−4.039231	−90.8899	0.578023	0.107964	−1.0797	−1.32031	−0.20194
B.C	0.003383	0.0662	−0.000113	−0.000005	0	−0.00014	−0.00004
B.D	0.001237	0.0292	−0.003289	−0.000015	0.0003	0.00001	0.00016
B.E	0.000037	0.0009	−0.000039	−0.000001	0	0	0
B.F	−0.000424	−0.0100	−0.000157	−0.000002	0	0.00006	0
B.G	0.692701	4.9660	0.028213	−0.001768	0.0118	0.00415	−0.00149

B.H	−0.050349	4.1005	0.018952	−0.000162	−0.0057	−0.00955	−0.00210
C.D	0.055078	0.3176	−0.001365	−0.000025	0.0003	−0.00240	−0.00023
C.E	0.001386	0.0082	−0.000030	−0.000008	0	0.00005	0.00001
C.F	−0.015837	−0.0941	0.001434	0.000032	0	−0.00029	−0.00002
C.G	−2.116753	19.5851	0.130351	−0.005706	0.0176	0.02420	−0.01253
C.H	1.823739	17.8154	−0.044868	−0.003356	0.0043	−0.00785	−0.01113
D.E	−0.001184	0.0035	−0.000462	−0.000005	0	0	−0.00001
D.F	0.013527	−0.0404	−0.004137	−0.000164	0.0024	0.00227	0.00039
D.G	1.644937	15.3552	−0.032943	−0.002093	0.1362	0.07272	0.00546
D.H	−4.168237	−41.7288	0.244673	0.007135	−0.0314	0.16416	0.04854
E.F	0.000406	−0.0012	0.000028	0.000002	0	−0.00001	0
E.G	0.049348	0.4607	−0.005684	−0.000438	0.0082	0.00422	0.00132
E.H	−0.098387	−1.1224	0.001680	0.000011	0.0002	−0.00365	0.00065
F.G	−0.563978	−5.2646	−0.042009	0.002146	−0.0524	−0.03537	−0.01062
F.H	1.124427	12.8276	−0.056073	−0.003698	0.0057	0.05519	−0.00470
G.H	73.114755	−1854.4732	−11.734558	−0.316956	5.4530	9.38515	−0.22269

Table S2. Weight and bias between input layer and hidden layer ($BPNN_1$).

Term	LW01	LW02	LW03	LW04	LW05	LW06	LW07	LW08
Bias1	−0.0115	4.4193	−0.3722	0.1870	3.6246	0.9251	1.1954	2.2300
I1	0,0162	−0.9191	−0.9593	−0.0019	−0.8122	−0.0114	−0.0147	−0.0481
I2	−0.1088	2.1555	0.3239	0.0763	1.7791	−0.6050	0.1924	0.4202
I3	0.0145	0.7844	−0.0082	0.0114	0.7014	−0.1163	0.0370	−0.1068
I4	0.0182	0.0457	−0.0243	0.0025	0.0747	−0.0017	0.0083	0.0057
I5	0.0229	0.0222	−0.0251	0.0034	0.0326	0.0029	0.0081	0.0094
I6	−0.0100	−0.0285	0.0243	−0.0007	−0.0430	0.0004	−0.0083	−0.0094
I7	0.9363	1.6317	−0.5188	0.2421	1.3941	0.0032	0.7922	1.7902
I8	0.1200	0.1567	−0.9202	0.0579	0.0422	−0.0778	−0.2872	−0.6703

Table S3. Weight and bias between hidden layer and output layer ($BPNN_1$).

Term	OW01	OW02	OW03	OW04	OW05	OW06	OW07
Bias2	−1.1487	0.4542	−0.3564	−0.0274	0.1885	0.0189	−0.1157
LW01	−0.7271	0.7574	−0.1560	−0.0534	1.0052	0.1245	0.1017
LW02	0.1495	−1.3397	0.1037	0.0270	−0.0508	0.0103	0.0382
LW03	0.5722	0.0408	0.1710	0.0578	0.0037	−0.2180	−0.0289
LW04	2.0343	−2.2710	0.4902	−0.0277	0.6946	−0.0820	0.0845
LW05	−0.7382	1.0419	−0.3758	0.0283	0.0452	0.1608	0.0061
LW06	−0.7243	0.8132	−0.8419	0.0515	−0.0595	−0.1709	−0.0557
LW07	3.7497	−0.4517	0.3777	−0.0110	−0.2276	−0.1455	−0.0330
LW08	−1.6346	−0.0416	−0.1366	0.0043	0.0904	0.0485	−0.0069

Table S4. Weight and bias between input layer and hidden layer ($BPNN_2$) for Dim1 prediction.

Term	LW01	LW02	LW03	LW04	LW05	LW06	LW07	LW08
Bias1	−0.7468	0.3184	0.0573	−0.2186	0.3463	0.8486	0.0132	0.8802
I1	−0.5113	0.1047	−0.0316	0.4027	0.1993	−0.4599	0.3319	−0.4308
I2	−0.1853	−0.1203	−0.0433	−0.5106	0.9391	1.0791	−0.2795	0.7238
I3	−0.2639	−0.3525	0.4638	−0.1668	0.5566	0.6589	−0.0297	0.1610
I4	−0.1053	0.4661	−0.1645	−0.8845	−0.0311	−0.5038	−0.1787	−0.1926
I5	−0.3390	−0.5927	0.1499	−0.1913	0.6231	0.6757	0.2161	0.2834
I6	0.0774	−0.3079	0.0079	0.1199	−0.0902	0.0132	−0.3906	0.0105
I7	−0.2488	0.0876	−0.4514	−0.1115	0.3652	0.0752	−0.4278	−0.1039
I8	0.0074	0.0237	0.1092	−0.1101	0.0709	−0.0559	−0.0041	−0.1968

Table S5. Weight and bias between hidden layer and output layer ($BPNN_2$) for Dim1 prediction.

Term	OW01
Bias2	0.2547
LW01	−0.8602
LW02	0.7260
LW03	−0.7130
LW04	0.6339
LW05	−0.7852
LW06	0.9200
LW07	0.4977
LW08	0.9989

Table S6. Weight and bias between input layer and hidden layer ($BPNN_3$).

Term	LW01	LW02	LW03	LW04	LW05	LW06	LW07	LW08	LW09	LW10	LW11	LW12
Bias1	−1,2543	−1,3880	−0,0124	−0,3607	0,1781	−0,0755	0,2098	1,1296	−0,1348	1,6098	1,5830	0,0565
I1	−0,3269	0,9552	0,1122	0,3297	−0,1491	0,0976	−0,0316	0,6315	−0,1026	−1,1035	−0,8216	0,7732
I2	0,4811	−0,9875	−0,1151	−0,4785	0,5669	0,9545	−0,4838	−0,6665	−3,6050	0,8587	0,5052	−0,3642
I3	0,2651	−0,7841	−0,0069	0,7101	0,0327	−1,1708	−0,7095	0,0840	−0,7548	0,6409	0,6541	−0,1354
I4	−1,3950	−0,5356	0,1008	0,0137	−0,0071	0,1714	−0,5262	0,0143	3,3102	0,4920	−0,0254	−0,3212
I5	0,0249	−0,2346	0,7613	0,0989	0,0566	0,0500	0,1705	0,0247	0,0943	0,1815	0,1193	0,3630
I6	0,0328	0,2395	0,1816	−0,3913	−0,0010	0,2786	0,1681	−0,0634	0,0574	−0,1519	−0,2128	−0,7780
I7	−0,0426	0,2393	−0,1839	−0,1436	−0,1767	0,8098	−0,2985	0,3657	−0,9702	−0,5392	0,6421	0,1685
I8	−0,1147	−0,3548	0,0840	0,2869	0,1282	−0,2117	−0,0813	0,0310	−0,1684	0,4266	−0,0721	−0,3746

Term	LW13	LW14	LW15	LW16	LW17	LW18	LW19	LW20	LW21	LW22	LW23	LW24
Bias1	0,1636	0,5309	−2,8384	0,2026	−0,6618	−0,6477	0,5657	0,6819	0,7306	1,0678	−0,2685	0,0245
I1	0,0756	−0,5003	2,1556	−0,1452	0,3302	−0,0102	0,3638	−0,1280	0,3967	0,0014	−0,7218	−0,0079
I2	0,4360	−0,0199	−1,9842	−0,4607	0,3730	−0,2947	0,1475	−0,1728	−0,5981	−0,4017	0,1238	−0,0149

I3	−0,4449	0,1943	−0,9802	0,2778	0,0897	0,0448	0,3416	−1,1086	0,0884	−0,4226	0,2150	−0,0095
I4	0,0530	−0,3686	0,1260	−0,1340	−0,4532	0,6038	0,5780	−0,6137	−0,0068	−0,2781	−0,0468	0,0004
I5	−0,0430	−0,2124	−0,0250	−0,1812	0,1171	0,1529	0,0792	0,2253	0,0572	−0,0929	0,1071	−0,0189
I6	0,0543	0,1252	0,1330	0,0351	0,0102	−0,0421	−0,0993	0,1157	−0,0694	0,0647	0,0005	0,0020
I7	0,1242	−0,8582	−2,4138	0,3328	0,0770	0,6865	−0,0841	1,8125	0,8775	1,3048	−0,6705	−0,6165
I8	0,0738	0,5501	0,1396	0,1223	0,1115	−0,0646	0,1276	0,0581	0,1738	0,6877	−0,5183	−0,0973

Table S7. Weight and bias between hidden layer and output layer ($BPNN_3$).

Term	OW01	OW02	OW03	OW04	OW05	OW06	OW07
Bias2	0,1693	0,3751	0,8799	−0,0530	0,0030	0,0933	−0,1650
LW01	−0,0443	−0,2341	0,5504	0,0868	−0,0077	0,1164	−0,3714
LW02	−0,0268	−0,8885	−0,1363	0,0367	−0,0008	−0,3536	0,0838
LW03	−0,0254	0,2393	−0,5063	−0,6206	0,0147	0,5418	0,6848
LW04	−0,3670	−0,0787	−0,3858	−0,0244	−0,0068	0,0920	0,0128
LW05	0,9214	−0,8435	1,3868	−0,1641	0,0274	0,3423	0,2969
LW06	−0,2380	−0,0618	0,0545	−0,0846	−0,0059	−0,0401	0,0496
LW07	−0,1343	−0,3862	−0,0891	−0,0428	0,0195	0,2156	0,1816
LW08	−1,0093	−0,0151	−0,1172	0,1262	−0,0072	−0,1321	−0,3045
LW09	0,0087	0,0189	0,6335	0,0618	−0,0033	−0,1098	−0,1159
LW10	−0,0354	−1,0141	−0,3032	0,0653	−0,0039	−0,2953	0,0147
LW11	−0,3333	−0,5153	0,0669	0,1178	−0,0042	−0,0877	0,1183
LW12	0,0925	0,1758	0,0201	−0,1146	0,0026	0,0798	0,1400
LW13	0,1571	0,8769	−0,5865	−0,0342	0,0005	−0,1625	−0,0533
LW14	−0,1283	0,4491	−0,2318	−0,0091	−0,0075	−0,0417	−0,0212
LW15	−0,2674	−0,0594	0,1815	−0,0254	−0,0038	−0,0026	0,0663
LW16	0,2757	0,5436	0,0232	0,1356	0,0043	−0,0610	−0,1840
LW17	0,0980	0,3688	0,5013	0,0260	−0,0307	−0,3305	−0,0511
LW18	0,0786	0,5214	−0,1702	−0,0226	0,0090	0,0794	0,2416
LW19	−0,1311	−0,6705	0,0774	−0,0781	−0,0011	0,3502	0,0669
LW20	−0,1947	−0,3626	0,0778	−0,1602	0,0098	0,2323	0,0729
LW21	1,0104	0,3322	−0,4763	−0,0908	0,0251	0,3955	0,1855
LW22	−0,2198	0,3308	−0,1879	−0,0381	−0,0036	−0,2909	0,0108
LW23	0,2133	0,5620	−0,4426	−0,0670	0,0000	−0,0267	0,1786
LW24	−0,3170	0,7958	−0,4804	0,0309	−1,5373	−0,1368	−0,1699
